# Molecularly engineered carrier-free co-delivery nanoassembly for self-sensitized photothermal cancer therapy

**DOI:** 10.1186/s12951-021-01037-6

**Published:** 2021-09-20

**Authors:** Xinzhu Shan, Xuanbo Zhang, Chen Wang, Zhiqiang Zhao, Shenwu Zhang, Yuequan Wang, Bingjun Sun, Cong Luo, Zhonggui He

**Affiliations:** Department of Pharmaceutics, Wuya College of Innovation, Shenyang Pharmaceutical University, Shenyang, 110016 People’s Republic of China

**Keywords:** Photothermal photosensitizers, Thermoresistance, HSP90 inhibitor, Dual-drug nanoassembly, Self-sensitized photothermal therapy

## Abstract

**Background:**

Photothermal therapy (PTT) has been extensively investigated as a tumor-localizing therapeutic modality for neoplastic disorders. However, the hyperthermia effect of PTT is greatly restricted by the thermoresistance of tumor cells. Particularly, the compensatory expression of heat shock protein 90 (HSP90) has been found to significantly accelerate the thermal tolerance of tumor cells. Thus, a combination of HSP90 inhibitor and photothermal photosensitizer is expected to significantly enhance antitumor efficacy of PTT through hyperthermia sensitization. However, it remains challenging to precisely co-deliver two or more drugs into tumors.

**Methods:**

A carrier-free co-delivery nanoassembly of gambogic acid (GA, a HSP90 inhibitor) and DiR is ingeniously fabricated based on a facile and precise molecular co-assembly technique. The assembly mechanisms, photothermal conversion efficiency, laser-triggered drug release, cellular uptake, synergistic cytotoxicity of the nanoassembly are investigated in vitro. Furthermore, the pharmacokinetics, biodistribution and self-enhanced PTT efficacy were explored in vivo.

**Results:**

The nanoassembly presents multiple advantages throughout the whole drug delivery process, including carrier-free fabrication with good reproducibility, high drug co-loading efficiency with convenient dose adjustment, synchronous co-delivery of DiR and GA with long systemic circulation, as well as self-tracing tumor accumulation with efficient photothermal conversion. As expected, HSP90 inhibition-augmented PTT is observed in a 4T1 tumor BALB/c mice xenograft model.

**Conclusion:**

Our study provides a novel and facile dual-drug co-assembly strategy for self-sensitized cancer therapy.

**Graphic abstract:**

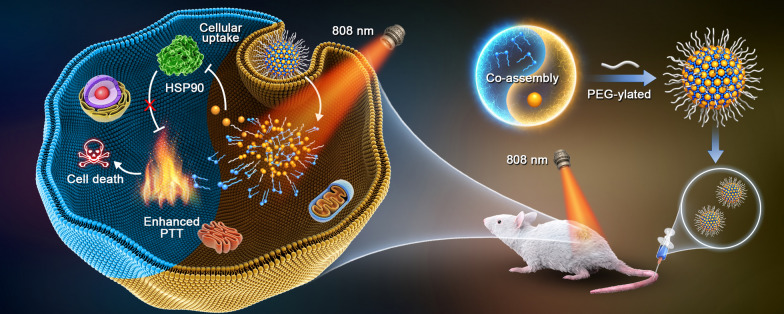

**Supplementary Information:**

The online version contains supplementary material available at 10.1186/s12951-021-01037-6.

## Background

Cancer is still a severe challenge to human health [1]. Conventional cancer therapeutics such as surgery, chemotherapy and radiotherapy still fall short of expectation owing to their own defects [2, 3]. Development of new therapeutic strategies has long been priority [4, 5]. Photothermal therapy (PTT) has attracted considerable attention as a promising non-invasive therapeutic modality with spatio-temporal selectivity and high security [6–9]. Series of organic photothermal agents have been found to exert antitumor activity by converting the near-infrared (NIR) light into tumor-localized heat [10, 11]. Moreover, fluorescence characteristics in most photosensitizers (PSs) endows them a natural advantage in real-time tumor imaging and therapeutic monitoring [12–14]. In other words, PTT represents a versatile therapeutic regimen for image-guided precision cancer treatment [6, 15–18].

Despite of all these advantages, PTT alone is usually unable to completely eradicate tumors, including 4T1 breast tumors [11, 15, 19–22]. One of the major reasons should be the cytoprotective pathways towards hyperthermia activated in tumor cells under laser irradiation, which inducing the overexpression of heat shock proteins (HSPs) under thermal stimulation [19, 23]. There is growing evidence that the overproduced HSPs participate in the repair of thermal damage to proteins, and enhance the stress reaction and thermotolerance of tumor cells [24, 25]. According to the size of proteins, HSPs are divided into five categories: HSP110, Hsp90, HSP70, HSP60 and small heat shock proteins. Among them, HSP90 has been found as an important molecular chaperone involving in the regulation of functional signaling proteins related to tumor growth and progression and shows high expression in tumor site [26]. Based on this rationale, various HSP90 inhibitors have been widely employed to increase the therapeutic sensitivity of tumor cells to PTT [27, 28].

Among them, gambogic acid (GA, a natural product) has been found to have potent inhibitory action on HSP90 [23]. It is reasonable that the combination of GA and photothermal PSs could significantly improve the PTT efficacy by surmounting the thermoresistance of tumor cells. Nevertheless, it’s still technically challenging to synchronously delivery two therapeutic agents towards tumor tissues [29–31]. Over the past few decades, multitudinous nano-vehicles have been designed for simultaneous co-encapsulation and co-delivery of two or more different drugs. However, these conventional co-delivery nanocarriers still have many drawbacks, such as low encapsulating or co-loading efficiency, inconvenient regulation of drug proportions, and inconsistency drug release caused by the affinity difference between carrier materials and different drugs [12, 32]. Hence, there’s an urge need to develop more efficient co-delivery nanoplatforms.

**Fig. 1:**
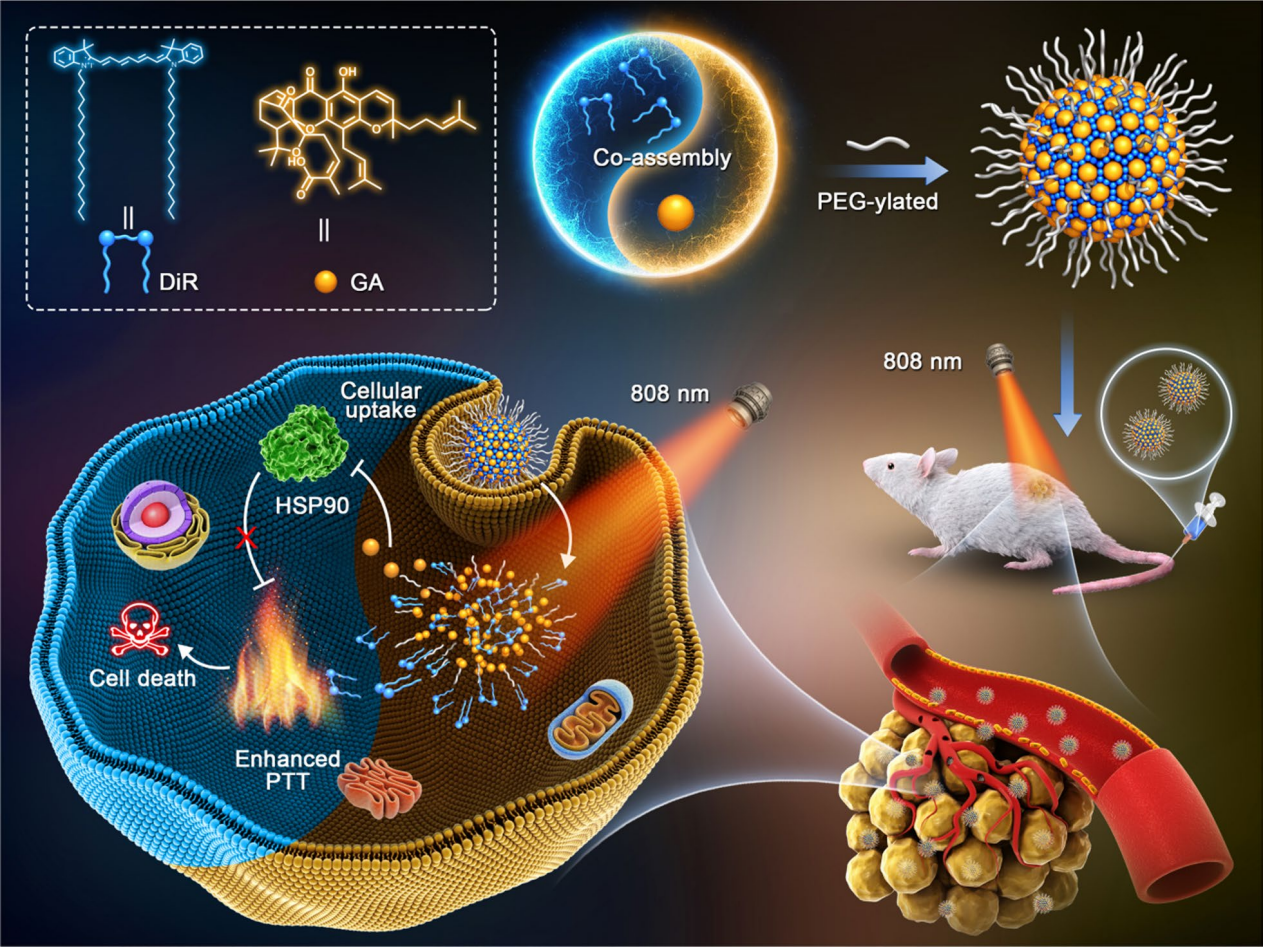
Schematic illustration of DiR/GA co-delivery nanoassembly and HSP90 inhibition-enhanced PTT by hyperthermia sensitization

We proposed that the hydrophobic GA molecule with aromatic groups and double bonds might perform nanoassembly with certain hydrophobic photothermal PSs. Interestingly, 1,1′-dioctadecyl-3,3,3′,3′-tetramethylindotricarbocyanine iodide (DiR, a commonly used photothermal agent) was found to be able to co-assemble with GA in aqueous environment (Fig. 1). Based on such unique molecular co-assembly feature, a precisely engineered carrier-free nanoassembly of DiR and GA was fabricated with the optimal synergistic dose ratio (3:1). This is the first attempt to precisely co-deliver HSP inhibitor and photothermal PSs based on pure drug-engineered assembly technique. This uniquely carrier-free nanosystem demonstrated distinct advantages throughout the whole drug delivery process, showing excellent HSP inhibition-facilitated PTT effect in 4T1 tumors bearing mice. This work provided a novel imaging-guided theranostic nano-platform and optimized the existing PTT approach with promising clinical transformation potential.

## Results and discussion

### Rational design and optimization of co-delivery nanoassembly

**Fig. 2:**
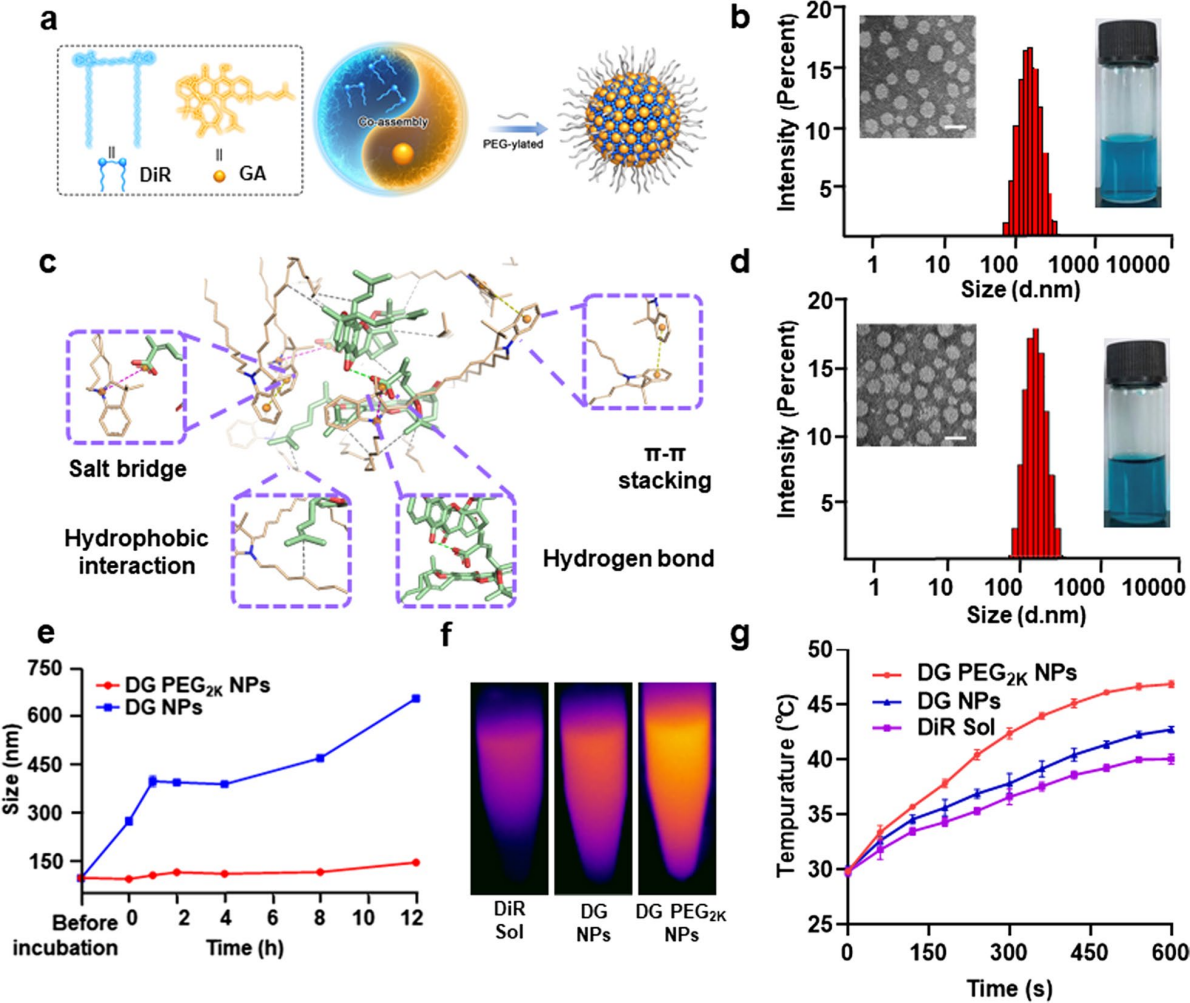
Characterization of the hybrid nanoassemblies. **a** Schematical illustration of the preparation of DG PEG_2K_ NPs; **b** Intensity size distribution profiles, TEM images as well as intuitive photographs of DG NPs, the scale bar represents 100 nm; **c** Molecular dynamics simulation of DiR and GA molecules in hybrid nanoassembly; **d** Intensity size distribution profiles, TEM images as well as intuitive photographs of DG PEG_2K_ NPs, the scale bar represents 100 nm; **e** Colloidal stability of DG NPs and DG PEG_2K_ NPs during incubation in PBS (pH = 7.4) (n = 3); (**f**, **g** In vitro photothermal efficiency of DiR Sol, DG NPs and DG PEG_2K_ NPs after incubated in PBS (pH 7.4) for 12 h (n = 3)

Hybrid nanoassemblies were fabricated by one-step nano-precipitation technique, with various molar ratios of 1:1, 2:1, 3:1, 5:1, 7:1 and 10:1 (DiR:GA). The mean diameters of above formulations were around 100 nm with PDI values almost less than 0.2 (Additional file 1: Tables S1 and S2). These results indicated that DiR and GA showed good co-assembly performance at a wide range of dose ratio. Then, the synergistic cytotoxic effect was explored to screen the optimal nano-formulation. As shown in Additional file 1: Table S3 and Fig. S1, the hybrid nanoassembly of DiR and GA at the molar ratio of 3:1 (DiR: GA) showed the most potent cytotoxicity against 4T1 cells with a CI value of 0.371, thus selected as the final formulation utilized in the following studies. As shown in Fig. 2b, the as-fabricated hybrid DiR/GA nanoassembly, denoted as DG NPs, revealed regular spherical structure with uniform particle size of around 90 nm according to the TEM images and the dynamic light scattering (DLS) results.

Then, molecular docking technique was applied to investigate the co-assembly mechanisms of DiR and GA. As shown in Fig. 2c, multiple intermolecular forces were found in this nanosystem, including hydrophobic interaction, salt bridge, π-π stacking interaction and hydrogen bonds. Besides, some confirmatory tests were also carried out to verify the existence of these aforementioned intermolecular interactions. The DG NPs was incubated with urea, NaCl and SDS which served as the interaction breakers of hydrogen bond, salt bridge and hydrophobic force, respectively. The diameters of DG NPs increased up to 4.99-folds, 1.49-folds and 12.67-folds after incubation with the above interaction breakers, suggesting the dominate role of hydrophobic interaction in this nanosystem (Additional file 1: Fig. S3).

To enhance the colloidal stability and prolong the systemic circulation time of the hybrid nanoassembly, DSPE-PEG_2k_ was decorated on the surface of nanoassembly. The PEGylated nanoassembly, denoted as DG PEG_2K_ NPs, showed similar size with DG NPs (Fig. 2d), with smaller PDI value of 0.128, indicating the narrow diameter distribution of DG PEG_2K_ NPs (Additional file 1: Table S2). The drug loading rate of the DG PEG_2K_ NPs was up to 66.1% of DiR and 13.9% of GA, suggesting excellent drug delivery advantages. Moreover, the ultraviolet absorbance spectrum of DG NPs and DG PEG_2K_ NPs appeared a slight red-shift when compared to that of DiR Sol, demonstrating the existence of π-π stacking in this hybrid nanoassembly (Additional file 1: Fig. S4).

We then investigated the colloidal stability of DG NPs and DG PEG_2K_ NPs by incubating them with PBS (pH = 7.4) at 37 °C. As shown in Fig. 2e, a sharp size increase of DG NPs up to 700 nm was observed in the presence of PBS, while the DG PEG_2K_ NPs maintained good stability under the same conditions. Moreover, DG PEG_2K_ NPs showed good stability in the presents of 10% FBS supplemented PBS under pH 6.6, 7.0, 7.4, 7.8 (Additional file 1: Fig. S5). The excellent colloidal stability of DG PEG_2K_ NPs should be attributed to the interparticle steric repulsion of PEGylated decoration.

### In vitro photothermal conversion efficiency

DiR has been widely applied for antitumor PTT. To explore whether the co-assembly process would produce an influence on the photothermal efficiency of DiR, the temperature variation of DiR Sol, DG NPs and DG PEG_2K_ NPs were investigated under a 808 nm laser irradiation every 30 s within 300 s. As shown in Additional file 1: Fig. S6, DiR Sol, DG NPs and DG PEG_2K_ NPs presented rapid temperature rising up to 55 °C. Significant apoptosis of tumor cells could be induced at this temperature [14]. In comparison, the temperature of PBS seemed to be almost unchanged under the same conditions (Additional file 1: Fig. S6). Moreover, the photothermal conversion efficiency (PCE) of DiR Sol, DG NPs and DG PEG_2K_ NPs were measured and calculated as 30, 31.2 and 31.7%, respectively (Additional file 1: Fig. S7). Though inferior to some inorganic photothermal materials, such as carbon-based NPs (around 25–80%) [33, 34] and gold NPs (around 30–60%) [35–37], the PCE of DiR and nanoassemblies still demonstrated significant advantage over most organic PSs, such as ICG (~ 15%) and IR780 (~ 19%) [34, 38]. These results demonstrated that the co-assembly process exerted negligible influence on the PCE of DiR.

Besides, the photothermal efficacy of DiR Sol, the DG NPs and DG PEG_2K_ NPs after incubation in PBS (pH 7.4) was measured. As shown in Fig. 2f-g, although DiR Sol, DG NPs and DG PEG_2K_ NPs had similar photothermal efficiency in pure water, DG PEG_2K_ NPs exhibited higher photothermal efficiency than DiR Sol and DG NPs after incubation in PBS for 12 h, which could be attributed to its good stability in PBS (Fig. 2e). By contrast, the incubation with PBS could result in the aggregation and even precipitation of DiR molecules in DiR Sol and DG NPs, thus decreasing their photothermal efficiency.

### In vitro light-triggered drug release

It was expected that the photothermal effect produced by DiR under laser irradiation could promote drug release from DG PEG_2K_ NPs. Thus, we evaluated the GA release performance from DG PEG_2K_ NPs with and without laser irradiation. As shown in Additional file 1: Fig. S8, GA could be rapidly released from GA Sol, while DG PEG_2K_ NPs exhibited slower drug release rate than GA Sol. Interestingly, GA release from DG PEG_2K_ NPs significantly increased when compared to the group of DG PEG_2K_ NPs without laser treatment, which could be probably attributed to the photo-induced disintegration of nanoassembly. Light-triggered drug release within tumor tissues could significantly enhance the HSPs inhibition of GA and reduce its systemic toxicity.

### Cellular uptake and synergistic photothermal cytotoxicity

**Fig. 3:**
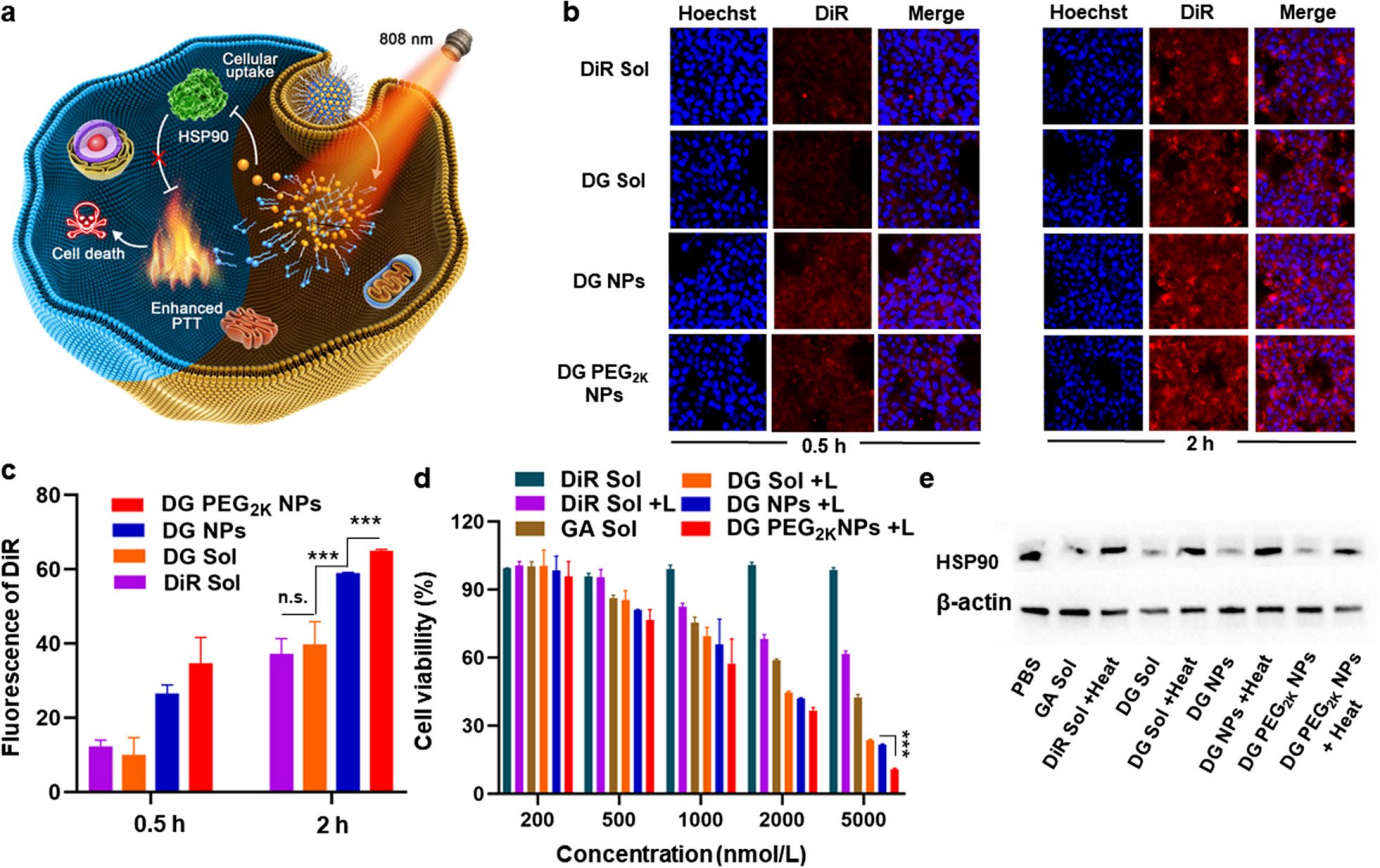
Cellular uptake and synergistic in vitro antitumor effect of hybrid nanoassemblies. **a** Intracellular mechanism of HSP90 inhibition -enhanced PTT by hyperthermia sensitization; **b** Confocal imaging of 4T1 cells treated with DiR Sol, DG Sol, DG NPs and DG PEG_2K_ NPs for 0.5 and 2 h, at a DiR equivalent dose of 2 μmol/L; **c** Quantitative results of cellular uptake in 0.5 and 2 h from flow cytometry, respectively; **d** Synergistic cytotoxicity against 4T1 cells with or without laser irradiation (3 W/cm^2^, 3 min); **e** Western blotting analysis of HSP90 protein expression in 4T1 cells with simulated heat treatment at 50 °C for 3 min. Scale bar = 50 μm. n.s. no significance, ***P < 0.001

DiR and GA lay a valid intracellular synergistic mechanism for potent PTT against tumors (Fig. 3a). Therefore, it is of great significance to study the intracellular fate of the hybrid nanoassemblies and their influence on tumor cells. The intracellular fluorescence signals of DiR monitored by CLSM and flow cytometry were employed to evaluate the cellular uptake of the hybrid nanoassembly in 4T1 cells. As shown in Fig. 3b, the fluorescence intensity in cells treated with DiR Sol, DG Sol, DG NPs and DG PEG_2K_ NPs significantly enhanced along with the extension of incubation time from 0.5 to 2 h under CLSM. Notably, DG NPs and DG PEG_2K_ NPs showed higher cellular uptake efficiency than that of DiR Sol or DG Sol in both 0.5 and 2 h. The flow cytometry results were consistent with the CLSM images (Fig. 3c and Additional file 1: Fig. S9). These results indicated that NPs had higher cellular uptake efficiency than that of drug solutions due to the endocytosis mechanisms of particles.

As previously mentioned, the photothermal effect produced by DiR could lead to the compensatory overexpression of intercellular HSP to protect against hyperthermal cell damage. The inhibition of HSP90 by GA could improve the hyperthermia sensitization to tumor cells [23, 24]. The synergistic chemo-photothermal cytotoxicity of hybrid nanoassembly was evaluated at the optimal molar ratio of 3:1 (DiR: GA) with or without laser irradiation. As shown in Fig. 3d and Additional file 1: Fig. S10, DG Sol, DG NPs and DG PEG_2K_ NPs presented significant cytotoxicity under laser irradiation, suggesting the synergistic anticancer effects of the two drugs. Notably, DG PEG_2K_ NPs presented more potent cytotoxicity than DG Sol and DG NPs under 808 nm laser irradiation (Additional file 1: Fig. S10 and Table S5), which should be ascribed to its favorable colloidal stability, photothermal efficiency and cellular uptake. Additionally, the increased fluorescence of lipophilic DiR molecules anchored on the membrane could also decrease its PTT efficiency. These results suggested that precisely formulating GA and DiR into carrier-free nanoassembly could achieve HSP inhibition-mediated PTT sensitization.

### In vitro mechanisms of self-sensitized PTT

Encouraged by the excellent synergistic cytotoxicity, we were eager to verify the synergistic mechanisms of self-sensitized PTT. Western blotting assay was carried out to determine the inhibitory effect of GA on HSP90, which is usually over-expressed when PTT-related hyperthermia in tumor cells (Fig. 3e and Additional file 1: Fig. S11). The expression of HSP90 was significantly up-regulated in the cells treated with heat. GA Sol could downregulate the cellular expression of HSP90 when compared with the PBS group, suggesting the inhibitory capacity of GA on HSP90. Notably, an obvious downregulation of HSP90 were found in the cells receiving DG Sol, DG NPs and DG PEG_2K_ NPs with and without heat when compared with the PBS-treated group. In comparison, DG PEG_2K_ NPs exhibit more significant inhibition effect compared with DG Sol and DG NPs. The excellent HSP90 inhibitory effect of DG PEG_2K_ NPs should be ascribe to its good colloidal properties and efficient cellular uptake. These results indicated that GA has the ability to inhibit the cellular expression of HSP90, and formulating it into NPs can further improve the inhibition effect.

### Pharmacokinetics

**Fig. 4:**
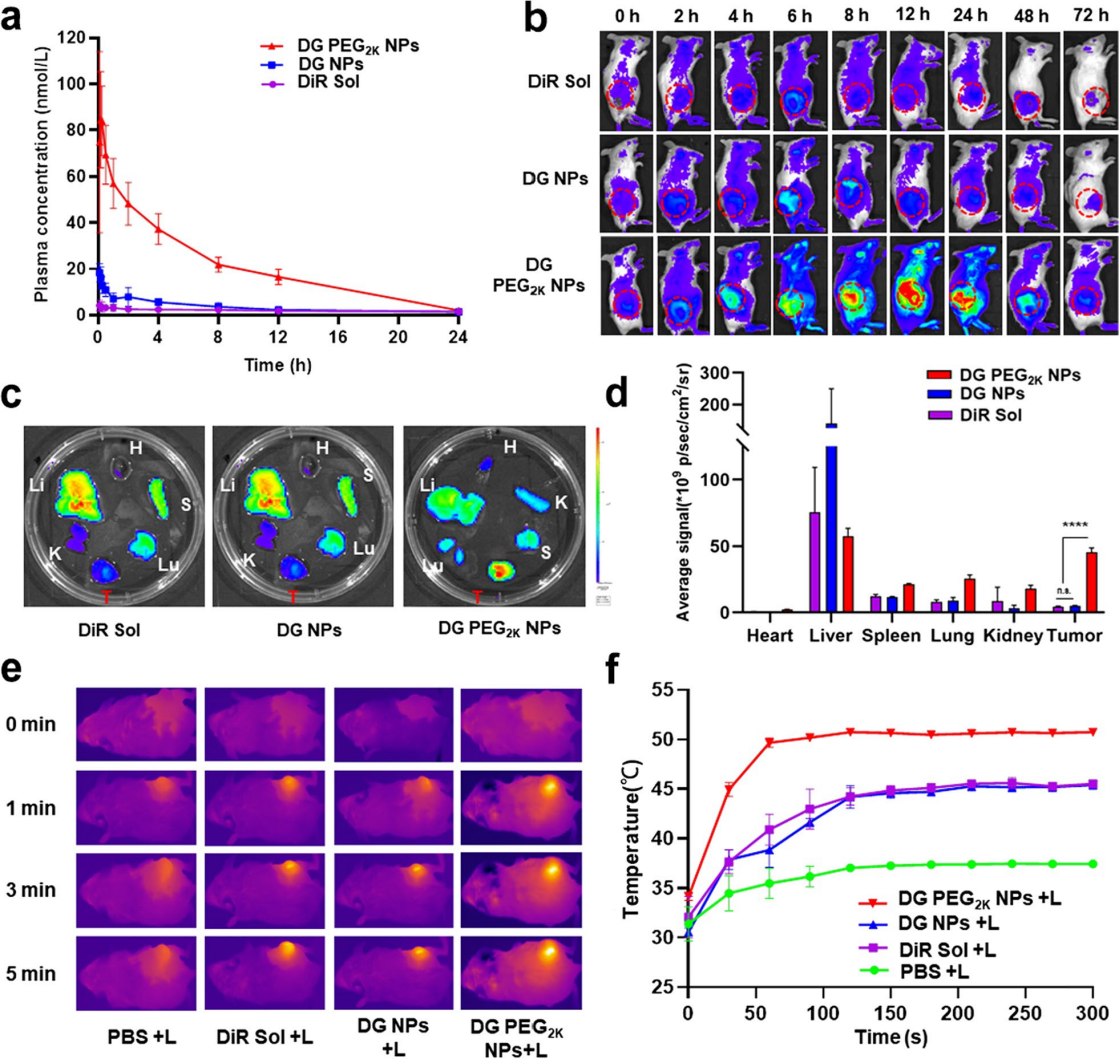
The pharmacokinetic profile and in vivo photothermal efficiency of DG PEG_2K_ NPs. **a** The concentration–time curves of DiR Sol, DG NPs and DG PEG_2K_ NPs at a DiR equivalent dose of 1 mg kg^−1^ (n = 6); **b** Living image of 4T1 tumor-bearing BALB/c mice treated with DiR Sol, DG NPs and DG PEG_2K_ NPs at a DiR equivalent dose of 1 mg/kg (n = 3), the tumor is indicated by circles; **c** Ex vivo image of major organs and tumors at the peak point of tumor accumulating (6 h post-injection for DiR Sol and DG NPs and 12 h for DG PEG_2K_ NPs); **d** Average fluorescence intensity of **c** ( n.s. no significance, ****P < 0.0001.); **e** Thermal imagery of tumor-bearing mice model after treated with PBS, DiR Sol, DG NPs and DG PEG_2K_ NPs; **f** The corresponding temperature changing curves of photothermal imaging in tumor site under laser treatment (808 nm, 3 W/cm^2^, 5 min)

The favorable colloidal stability, photothermal efficiency, cellular uptake performance, synergistic cytotoxicity with exact mechanisms made DG PEG_2K_ NPs a promising candidate for further investigation in vivo. The systemic circulation time of therapeutic agents in the blood exerts an important influence on the in vivo delivery destiny and therapeutic outcome of drugs. The pharmacokinetic behaviors of DiR Sol, DG NPs and DG PEG_2K_ NPs were investigated in SD rats. The plasma concentrations of DiR were determined by fluorescence analysis. As shown in Fig. 4a, DiR Sol was quickly cleared from blood circulation after intravenous injection. DG NPs with hydrophobic surface also showed poor pharmacokinetic behavior, which could be ascribed to its poor colloidal stability and the RES-mediated rapid clearance in the body. As expected, DG PEG_2K_ NPs demonstrated significant advantages in terms of systemic circulation time over DiR Sol and DG NPs, profiting from favorable colloidal stability and the stealth effect after PEG-decoration.

Delivery efficiencies of nanomedicine depended on passive tumor targeting ability which is closely related to their blood circulation time and modulation effect of the tumor microenvironment. Excellent pharmacokinetic behavior may endow DG PEG_2K_ NPs with good tumor accumulation ability.

### Biodistribution

Excellent tumor accumulation will undoubtedly favor efficient PTT. According to the accumulation of NPs monitored by the fluorescence intensity changes of DiR in tumors, the optimum time for laser treatment in PTT could be well determined [39, 40]. To explore the tumor targeting accumulation ability of DG PEG_2K_ NPs and to figure out an optimal laser irradiation time for image-guided synergistic tumor treatment, the time-varying biodistribution of the nanoassembly were evaluated by detecting the fluorescence of DiR in tumor-bearing mice. First, an overall accumulation of nanoassembly in the body was carried out by living imaging. As shown in Fig. 4b, DG PEG_2K_ NPs presents significantly stronger fluorescence intensity than that of DiR Sol and DG NPs within 72 h after intravenous injection of these formulations. The in vivo imaging results were well consistent with the pharmacokinetic behaviors of DG PEG_2K_ NPs. Besides, DG PEG_2K_ NPs showed much higher tumor accumulation when compared to DiR Sol and DG NPs, which will be helpful for tumor-localized PTT. Moreover, the peak points of tumor fluorescence intensity were located at 6, 6, 12 h for DiR Sol, DG NPs and DG PEG_2K_ NPs, respectively. We then quantitatively examined the fluorescence intensity of these formulations in major organs and tumors at their peak points. As shown in Fig. 4c, d, DG PEG_2K_ NPs demonstrated distinctly strong fluorescence intensity in tumor tissues compared to DiR Sol and DG NPs. These results revealed that the PEGylated nanoassembly could be efficiently accumulated in tumor tissues due to its long circulation characteristics in the blood, and the biodistribution results provided a clear guidance for the proper laser irradiation time (DiR Sol and DG NPs at 6 h post-administration and DG PEG_2K_ NPs at 12 h post-administration).

### In vivo photothermal efficacy

Favorable in vivo photothermal efficiency is considered to be a key for obtaining satisfying photothermal antitumor effect. The in vivo photothermal efficiency of nanoassembly was evaluated in tumor bearing mice under laser irradiation. The thermal imagery and tumor local temperature elevation of tumor-bearing mice were recorded during laser irradiation at 6 h post-injection for PBS, DiR Sol and DG Sol and 12 h for DG PEG_2K_ NPs. As shown in Fig. 4e, f, DG PEG_2K_ NPs displayed higher temperature elevation at tumor site (over 50 °C) than that of DiR Sol and DG NPs (around 43 °C), suggesting high photothermal efficacy. The excellent in vivo photothermal efficacy could be attributed to its favorable pharmacokinetic behavior and efficient tumor accumulation capacity. Thus, DG PEG_2K_ NPs were expected to exert a highly effective photothermal antitumor efficacy.

### In vivo photothermal antitumor efficacy

**Fig. 5:**
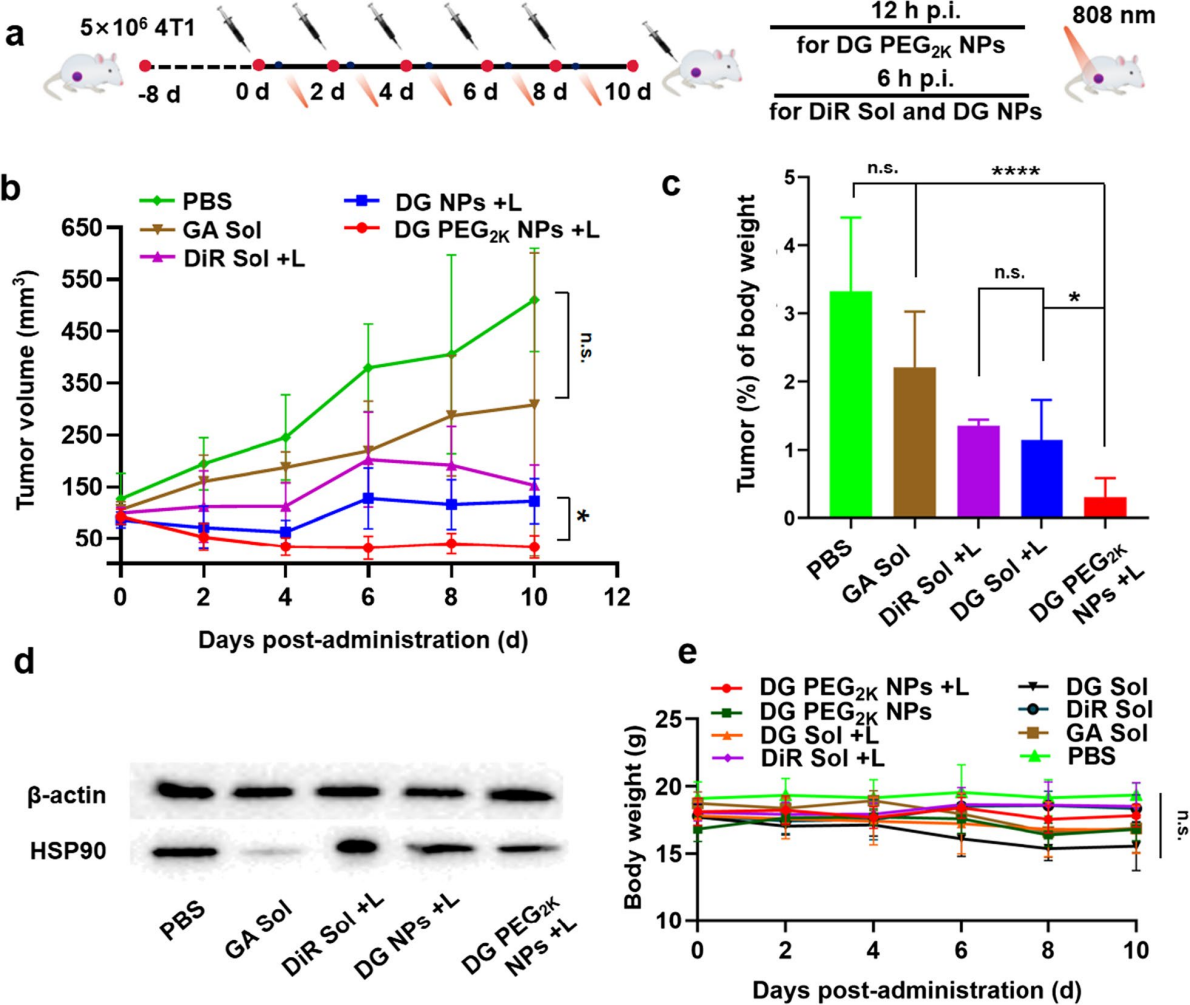
In vivo antitumor activity against 4T1 breast xenograft tumors (n = 5). **a** Illustration of the treatment schedule for HSP inhibition-potentiated PTT; **b** Tumor growth profiles; **c** Tumor % of body weight; **d** Western blotting results of HSP90 expression in tumor tissues; **e** Body weight changes of BALB/c mice bearing 4T1 tumors after injected with different formulations. n.s. = no significance, * P < 0.05, **** P < 0.0001

The satisfactory features and performance of the PEGylated nanoassembly inspired us to further evaluate the synergetic antitumor activity in 4T1 breast tumor-bearing mice. PBS, DiR Sol, GA Sol, DG Sol and DG PEG_2K_ NPs were intravenously administered to the mice every two days with a total 5 injections at doses equivalent to 5 mg/kg of DiR and/or 1.7 mg/kg of GA. DiR Sol and DG Sol groups received laser irradiation (808 nm, 3 W/cm^2^, 3 min) at 6 h post-administration and DG PEG_2K_ NPs were imposed irradiation at 12 h according to the biodistribution results (Fig. 5a). As shown in Fig. 5b–d and Additional file 1: S12–14, the PBS, DiR Sol without laser and GA Sol groups demonstrated a rapid increase in tumor volume, suggesting the inferior in vivo delivery characteristics of drug solutions. DiR Sol could partially suppress tumor growth to a certain degree under laser irradiation. DG Sol demonstrated more remarkable antitumor activity under laser irradiation when compared with the individual drug solutions (GA Sol and DiR Sol with laser treatment), indicating the synergistic performance between DiR and GA (Fig. 5b). As expected, DG PEG_2K_ NPs with laser treatment showed the most potent anti-tumor activity among these formulations, which could be ascribed to its good pharmacokinetic behavior, high tumor specific accumulation, favorable in vivo photothermal efficacy as well as HSP90 inhibition-augmented PTT (Fig. 5b, c).

Moreover, the results of Ki67 staining and TUNEL assay also confirmed the potent tumor inhibiting capacity of DG PEG_2K_ NPs, with the smallest tumor proliferation and the strongest apoptosis (Additional file 1: Figs. S15 and S16). The in vivo synergistic mechanism of HSP90 inhibition-sensitized PTT was then verified by western blotting assay. As shown in Fig. 5d and Additional file 1: Fig. S17, tumors treated with DiR Sol and laser significantly facilitated the HSP90 expression, indicating the existence of PTT-induced thermoresistance in tumor cells. Notably, the expression of HSP90 in tumor tissues was greatly downregulated when treated with GA Sol, further confirming its efficient inhibition activity on HSP90. As expected, DG Sol and DG PEG_2K_ NPs effectively restrained the increasement of HSP90 expression under laser irradiation when compared with DiR Sol under the same conditions. These results were well consistent with the in vitro western blotting assay and provided a direct evidence for self-sensitized multimodal cancer therapy in basis of HSP90 inhibition.

In addition to therapeutic efficacy, therapeutic safety has a higher priority for the clinical translation and application of nanomedicines. The off-target toxicity of nanoassembly was evaluated in the course of treatment. As shown in Fig. 5e and Additional file 1: Fig. S18, no significant change in the body weight of tumor-bearing mice was found throughout the treatments. Moreover, there is no abnormality seen in the detection index of hematologic, hepatic and renal toxicity (Additional file 1: Fig. S18). Besides, H&E staining results also revealed that no distinct damage was revealed in major organs (heart, liver, spleen, lung and kidney) (Additional file 1: Fig. S19).

## Conclusions

Taken together, we rationally engineered a co-assembly of DiR and GA for self-sensitized multi-model cancer therapy by HSP90 inhibition-based PTT sensitization. Based on the screening results of synergistic cytotoxicity, a precision dual-drug nanoassembly was elaborately fabricated at the optimal molar ratio of 3:1 (DiR:GA). The PEGylated nanoassembly (DG PEG_2K_ NPs) showed comprehensive advantages throughout the drug delivery process, including (i) facile preparation with good reproducibility; (ii) long circulation in the blood and favorable accumulation in tumor tissues; (iii) excellent cellular uptake performance; (iv) efficient photothermal capacity; (v) laser triggered-drug release; and (vi) self-sensitized cancer therapy with definite synergistic mechanisms and high security. This is the first exploration of carrier-free nano-system integrating photothermal PSs and HSP90 inhibitor for self-sensitized antitumor photothermal therapy. Our work provides a novel and simple co-delivery strategy for multimodal tumor therapy with remarkable potential for clinical application.

## Methods

### Materials

DiR, GA, RPMI 1640 cell culture medium, phosphate buffered solution (PBS) pH = 7.4 and all the reagents applied in Western blotting assay were obtained from Meilun Biotech Co. Ltd. (Dalian, China). 1,2-distearoyl-sn-glycero-3-phosphoethanolamine-N-[methoxy(polyethyleneglycol)-2000] (DSPE-PEG_2K_) was purchased from AVT (Shanghai) Pharmaceutical Tech Co., Ltd, China. Penicillin–streptomycin and fetal bovine serum were acquired from GIBCO, Invitrogen Corp. (Carlsbad, California, USA). 3-(4,5-dimthyl-2-thiazolyl)-2,5-dipphenyl-2H-terazolium bromide (MTT) and trypsin–EDTA were acquired from Sigma-Aldrich, USA. Hoechst 33342 was obtained from BD Biosciences, USA. The anti-HSP90 antibodies were purchased from Abclonal Technology Co., Ltd. All the vessels for cell culture dishes were provided by Wuxi NEST Biotechnology Co., Ltd, China and any other solvents and chemicals were of analytical or HPLC grade.

### Screening the optimal synergistic dose ratio of DiR and GA

4T1 cells were cultured with RPMI 1640 contained penicillin (100 units/mL), streptomycin (100 μg/mL) and 10% fetal bovine serum (FBS) under the condition of 5% CO_2_ humidified atmosphere at 37 °C. Synergy of DiR in combination with GA was measured through MTT assay and calculated by median-effect equation of Chou-Talalay combination index (CI), which is a quantitative method that could accurately analyze the effect of drug compatibility (synergistic, antagonistic or additive effects) by using the formula[41] as follow:

$${\text{CI}} = {\text{ A }} \times {\text{DiR}}@{\text{GA}}_{{{\text{combine}}}} /{\text{DiR}}_{{{\text{alone}}}} + {\text{ B }} \times {\text{DiR}}@{\text{GA}}_{{{\text{combine}}}} /{\text{GA}}_{{{\text{alone}}}}$$

where “A” and “B” was the dose proportion of DiR and GA in the mixture of DiR and GA, respectively; DiR_alone_ and GA_alone_ refers to the calculated IC_50_ when the cells separately treated with DiR or GA; DiR @GA_combine_ was utilized to calculate the IC_50_ level when DiR was combination with GA. The outcomes could be divided into synergistic effect (CI < 1), additivity (CI = 1) and antagonistic effect (CI > 1).

In detail, 4T1 cells were seeded into 96-well plates as 3 × 10^3^ cells per well. After adherence, the culture medium was discarded and replaced by serial dilutions of DiR solution, GA solution or the DiR/GA mixture at molar ratios of 10:1, 7:1, 5:1 3:1 and 1:1, respectively. After incubation for 4 h, the laser-treated groups were exposed to an 808 nm laser irradiation (3 W/cm^2^, 3 min). Whereafter, the cells were sent back to the incubator for another 44 h cultivation before MTT assay. For MTT assay, 25 μL of MTT (5 mg/mL in PBS) was added to each well and further incubated for another 4 h at 37 ℃. Finally, the fluid in the wells was blotted up and substituted by 200 μL DMSO for the measurement of the UV absorbency of generated Formazan by Varioskan LUX multimode microplate reader (Thermo Scientific, USA) (n = 3).

### Construction and characterization of co-assembled DG NPs and DG PEG_2K_ NPs

One-step nano-precipitation method was adapted for construction of hybrid nano-assembly of DiR and GA. In detail, DiR and GA were firstly dissolved in ethyl alcohol (10 mg/mL), respectively. Then, a mixture of DiR solution (200 μL) and GA solution (42 μL) was dropwise added into deionized water (2 mL) under stirring for 3 min (1500 rpm), denoted as DG NPs. In order to further improve the colloidal stability of NPs, DSPE-PEG_2K_ was applied for surface modification on DG NPs, named as DG PEG_2K_ NPs. Specifically, 60.5 μL of DSPE-PEG_2K_ in ethyl alcohol (10 mg/mL) was rapidly dropped into the non-PEGylated DG NPs colloidal system. To end, the organic solvent was removed under vacuum condition at 35 °C.

The Zetasizer (Nano ZS, Malvern Co., UK) was utilized for characterizing the hydrodynamic size (polydispersity index Inc.) and Zeta potential of DG NPs and DG PEG_2K_ NPs. The morphologies were visualized by transmission electron microscope (TEM) (Hitachi, HT7700, Japan). Samples were stained by phosphotungstic acid (1%, w/v) prior to observation.

### Co-assembly simulation

Molecular docking simulation was adapted to explore the co-assembly details of DiR and GA. Moreover, the Autodock Vina software was utilized to construct the 3-dimensional structures of DiR and GA.

Besides, the existence of hydrophobic interaction, salt bridge interaction and hydrogen bond were validated by incubating DG PEG_2K_ NPs (100 μL) with SDS (100 nM, 5 mL), NaCl (100 nM, 5 mL) and urea (100 nM, 5 mL), respectively. The particle variation of DG PEG_2K_ NPs was measured by Zetasizer (Nano ZS, Malvern Co., UK) after incubation (n = 3).

### Colloidal stability

The changes of particle sizes were deemed to indicate colloidal stability of NPs. In brief, DG NPs and DG PEG_2K_ NPs were incubated in PBS (pH 7.4) under shake cultivation (37 ℃) for 12 h, respectively, and sizes of nanoassemblies at 0, 1, 2, 4, 6, 8 and 12 h were recorded to preliminarily evaluate the colloidal stability. Whereafter, DG PEG_2K_ NPs were incubated in 10% FBS supplemented PBS at pH of 6.6, 7.0, 7.4 and 7.8 under shake cultivation (37 °C) for 12 h, and the sizes of nanoassemblies at 0, 1, 2, 4, 6, 8 and 12 h were recorded as a further validation (n = 3).

### In vitro light-triggered drug release

The drug release behavior of DG PEG_2K_ NPs was determined by dialysis method. PBS (pH = 7.4) containing 30% of ethanol and 1% of Tween-80 was selected as the release medium. DG PEG_2K_ NPs and GA Sol (300 μL, both equivalent to 0.21 mg/mL GA) were tied up in dialysis bags, and the DG PEG_2K_ NPs with laser treatment group was exposed to 808 nm laser irradiation (3 W/cm^2^, 5 min). Then, all the drug contained dialysis bags were immersed in 30 mL release medium-contained centrifugal barrel in shaking table at 37 °C. At pre-set time points, 200 μL of release media were taken out and supplemented by equivoluminal fresh media. The cumulative release of GA from DG PEG_2K_ NPs was determined by HPLC. The chromatographic condition was as follows: C_18_ chromatographic column (4.6 × 150 mm, 5 μm); mobile phase: methyl alcohol: water (contained 1‰ acetic acid) = 70: 30. The flow rate was set to 1.0 mL/min; the wavelength was set to 360 nm for GA detection (n = 3).

### In vitro photothermal efficacy

The temperature rise under light irradiation was conducted to investigate the photothermal efficiency in vitro. Briefly, PBS, DiR Sol, DG NPs and DG PEG_2K_ NPs with a dilution of equivalent 0.2 mg/mL DiR was treated with 808 nm laser irradiation (2 W/cm^2^, 5 min). Moreover, DG NPs and DG PEG_2K_ NPs with a dilution of equivalent 0.2 mg/mL DiR with PBS was further treated with 808 nm laser irradiation (2 W/cm^2^, 5 min) after 12 h incubation. The temperature variation of the groups was recorded every 30 s by infrared thermal imaging camera (Fotric 226) (n = 3).

### Spectrum scanning

DiR Sol, DG NPs, DG PEG_2K_ NPs, and GA Sol (diluted to equal 25 μg/mL of DiR or 5.245 μg/mL of GA in advance) were added to 96-well plates, and the UV spectra were read by the multimode microreader.

### Cellular uptake and cytotoxicity

Cellular uptake assay was studied using 4T1 cells. The cells were seeded in 24-well plates padded with glass slide in advance as a density of 5 × 10^4^ cells per well. After incubation for 12 h, fresh culture media dilute with DiR Sol, mixed DiR and GA solution (denoted as DG Sol), DG NPs and DG PEG_2K_ NPs (both equivalent to 2 μg/mL of DiR and 0.42 μg/mL of GA) were introduced to replace original medium and cultured for 0.5 and 2 h, respectively. Later on, the drug-containing media were removed from the cells and further rinsing with ice-cold PBS for three times. For confocal laser scanning microscope imaging, the washed cell was fixed by 4% paraformaldehyde and stained by Hoechst 33,342 for 10 min to tint the nuclei. Finally, the intracellular fluorescence signals were observed by confocal microscopy (CLSM, C2, Nikon, Japan). For quantification by flow cytometry, the washed cells were digested by Trypsin, collected, centrifugated and sequentially resuspended in PBS for followed determination by FACS Calibur flow cytometer (n = 3).

The cytotoxicity of GA Sol, DiR Sol, DG Sol, DG NPs and DG PEG_2K_ NPs (with or without laser irradiation in each DiR contained groups) was evaluated by MTT assay in 4T1 cell line. 96-well plates containing adhered cells mentioned above were treated with fresh media containing serial concentrations of various aforementioned preparations, respectively (n = 3) The sequential operation including laser-irradiation for laser-treated groups, the adding of MTT solution and absorbency measurement to the generated formazan was completely in accordance with the method described above.

### Animal studies

All the animals adopted were in accordance with the requirements and regulations from the Animal Ethics Committee of Shenyang Pharmaceutical University.

### Pharmacokinetics studies

To explore the pharmacokinetic behavior, DiR Sol, DG NPs and DG PEG_2K_ NPs were injected into male Sprague–Dawley rats (180–220 g) through caudal vein with an equivalent DiR dose of 1 mg/kg, respectively (n = 6) At pre-set point-in-time of 0.083, 0.167, 0.25, 0.5, 1, 2, 4, 6, 8, 12 and 24 h, about 500 µL of blood were harvested from ophthalmic vein and centrifuged (1.3 × 10^4^ rpm, 3 min) to collect corresponding plasma samples. Then, all the plasma samples were treated by protein precipitation method to extract DiR from the plasma, and the concentrations of DiR in plasma were detected by multimode microreader (Thermo Scientific, USA) (n = 6).

### Biodistribution

4T1 tumor-bearing mice were employed as a model for biodistribution assay of the hybrid nanoassembly. Briefly, 100 μL of cells contained PBS (~ 10^7^ cells) were subcutaneously inoculated to female Balb/c mice. Once the tumor volume reached nearly 300 mm^3^, the mice were randomly divided into three groups. DiR Sol, DG NPs and DG PEG_2K_ NPs were intravenously injected to mice of each group via tail vein at an equivalent DiR dose (1 mg/kg). At predetermined time intervals (0, 2, 4, 6, 8, 12, 24, 48 and 72 h) post-injection, the mice were anesthetized and a semi-quantitative bio-distribution was performed by noninvasive optical imaging system with an exciation laser of 748 nm (IVIS Lumina Series III) (n = 3).

For quantitative analysis of major organs, the mice were sacrificed through cervical dislocation to acquire heart, liver, spleen, lung, kidney and tumors at the maximum tumor accumulation point of each group determined in in vivo imaging assay. The fluorescence intensity of organs and tumors were analyzed by noninvasive optical imaging system (IVIS Lumina Series III) (n = 3).

### In vivo photothermal efficacy

To explore the in vivo photothermal efficiency, DiR Sol, DG Sol, and DG PEG_2K_ NPs were intravenously treated to the 4T1 tumor bearing BALB/c mice at an equivalent dose of 5 mg/kg DiR, and PBS treated mice were regarded as control. At 12 h post-injection for the group of DG PEG_2K_ NPs and 6 h post-injection for the groups of DiR Sol, DG Sol and PBS, the tumor location was exposed to 808 nm laser irradiation (3 W/cm^2^, 3 min). The infrared thermographic images and variations of tumor local temperature were measured every 30 s in 5 min by infrared thermal imaging camera (Fotric 226) (n = 3).

### In vivo antitumor activity

The in vivo antitumor activity of hybrid nanoassemblies was explored on 4T1 tumor-bearing female Balb/C mice established as described previously. As the tumor volume reached 100 mm^3^, the mice were divided into 8 groups randomly (n = 5): PBS, GA Sol, DiR Sol, DiR Sol + L, DG Sol, DG Sol + L, DG PEG_2K_ NPs and DG PEG_2K_ NPs + L. The formulations was injected to the tumor-bearing mice intravenously every two days with equivalent doses of 5 mg/kg DiR and/or 1.05 mg/kg GA for total five treatments, respectively. Mice in laser-treated groups were exposed to laser L808 irradiation (3 W/cm^2^, 3 min) at 12 h post-injection for group of DG PEG_2K_ NPs + L and 6 h post-injection for groups of DiR Sol + L and DG Sol + L. The tumor volume as well as body weight were measured every two days. Two days after the last dose, the mice were sacrificed, blood samples were collected and the serum samples were further acquired through centrifugation (1.3 × 10^4^ rpm, 10 min) for hepatic and renal function analysis. Tumors and major organs (heart, liver, spleen, lung, kidney) were collected and dipped into 4% paraformaldehyde for evaluation of pathological changes via H&E staining. Moreover, Ki67 staining was carried out on tumor tissue for the terminal deoxynucleoitidyl transferase dUTP nick end labeling (TUNEL) assay.

### Western blotting assay

HSP90 was measured according to a previous work [25], total protein was extracted from cell samples and tumor tissues. For cell samples, DiR Sol, DG Sol, GA Sol, DG NPs and DG PEG_2K_ NPs contained culture medium were treated to cells as a concentration of 1 μmol/L DiR and/or 0.21 μmol/L GA, respectively. Additionally, we simulated the photothermal therapeutic effect by pretreating the preparations with NIR laser (808 nm, 3 W/cm^2^, 3 min) and incubating cells at 50 °C for 3 min. For tumor samples, the tumor tissues were extracted out from 4T1 tumor-bearing mice after final treatment for in vivo antitumor activity. Samples were qualified by BCA colorimetric method and equal amounts of protein-contained samples were scraped into SDS-PAGE electrophoresis and electro-transferred onto NC membrane (0.45 μm) subsequently. Later on, 5% defatted milk contained TBST (TBS contained 0.1% Tween 20) was utilized to block membranes at room temperature for 1 h. Next, the membranes were incubated with anti-HSP90 antibodies at 4 °C overnight and further incubated with the secondary antibodies at room temperature for 1 h, the protein bands were visualized by means of ECL Western Blotting Substrate. Further, HSP90 was quantified by ImageJ 1.51j8 (USA).

### Statistical analysis

The data were calculated and presented as mean value ± SD. T-test and one-way analysis of variance (ANOVA) were employed to analyze the significant differences. P < 0.05 was considered as statistically significant.

## Supplementary Information

**Additional file 1. **Additional methods, figures and tables.

## Data Availability

All data generated or analyzed during this study are included in this published article.

## Abbreviations

PTT: Photothermal therapy
PS: Photosensitizer
NIR: Near-infrared
HSPs: Heat shock proteins
GA: Gambogic acid
DiR: 1,1′-Dioctadecyl-3,3,3′,3′-tetramethylindotricarbocyanine iodide
PBS: Phosphate buffered solution
DSPE-PEG_2K_: 1,2-Distearoyl-sn-glycero-3-phosphoethanolamine-N-[methoxy(polyethyleneglycol)-2000]
PCE: Photothermal conversion efficacy
MTT: 3-(4,5-Dimthyl-2-thiazolyl)-2,5-dipphenyl-2H-terazolium bromide
FBS: Fetal bovine serum
CI: Combination index
PDI: Polydispersity index
TEM: Transmission electron microscope
CLSM: Confocal microscopy
H&E: Hematoxylin and eosin
DLS: Dynamic light scattering
EPR: Enhanced permeability and retention
