# Supplementary material for: Molecularly engineered carrier-free co-delivery nanoassembly for self-sensitized photothermal cancer therapy
Source: J Nanobiotechnology. 2021 Sep 20;19:282. doi: 10.1186/s12951-021-01037-6 (PMC8454134; doi:10.1186/s12951-021-01037-6)
Supplement: Supplementary file 1 — Additional file 1. Additional methods, figures and tables. [file 12951_2021_1037_MOESM1_ESM.docx]

**Additional file 1**

**Molecularly Engineered Carrier-free Co-delivery Nanoassembly for Self-sensitized** **Photothermal Cancer Therapy**

**Authors:** Xinzhu Shan^1a^, Xuanbo Zhang^1a^, Chen Wang^1^, Zhiqiang Zhao^1^, Shenwu Zhang^1^, Yuequan Wang^1^, Bingjun Sun^1^, Cong Luo^1*^, Zhonggui He^1*^

**Affiliations:**

^1^Department of Pharmaceutics, Wuya College of Innovation, Shenyang Pharmaceutical University, Shenyang 110016, PR China

^2^Department of Pharmacology, School of Life Science and Biopharmaceutics, Shenyang Pharmaceutical University, Shenyang, Liaoning 110016, PR China

^a^These authors contributed equally to this work.

***Corresponding authors:**

Cong Luo, Ph.D. and Zhonggui He, Ph.D.

Professors, Department of Pharmaceutics, Wuya College of Innovation, Shenyang Pharmaceutical University, 103 Wenhua Road, Shenyang 110016, China

Tel: +86-024-23986321; Fax: +86-024-23986321

E-mail address: luocong@syphu.edu.cn; hezhonggui@vip.163.com

**Additional method**

**Determination of photothermal conversion efficiency**

The photothermal conversion efficiency (PCE) of of DiR Sol, DG NPs and DG PEG_2K_ NPs were measured using Roper’s method [1]. Briefly, DiR Sol, DG NPs, and DG PEG_2K_ NPs with an equivalent DiR concentration of 0.3 mg mL^−1^ were added into an EP tube (1.5 mL), respectively. The above three samples were imposed irradiation by an 808 nm laser (MDL-N-5W, Changchun New Industries, China) at 2 W/cm^2^ for 10 min and then turned off laser to cool samples for 10 min. The temperature variations of these samples were determined using an infrared thermal imaging camera (Fotric 226).

The PCE (*η*) of DiR Sol was calculated by the following equation:

*η = hS(T_max_-T_surr_)-Q_dis_/ I^(1-10-A808 nm)^* (1)

where *h* is the heat transfer coefficient; *S* is the irradiated area; *I* represents the laser power density (2 W/cm^2^); *A_808 nm_* is the UV absorbance of the DiR Sol at 808 nm (3.338 for 0.3 mg/mL of DiR Sol); *T_max_* and *T_surr_* are maximum steady-state temperature and ambient temperature of the environment; *Q_dis_* is represents the heat dissipation from the light absorbed by solution. In equation (1), the value of *hS* is measured by the temperature reducing rate after turning off the light source, which could be calculated via the following equations:

*hS =mC_p_/* *τ_s_* (2)

t = -*τ_s_ ln θ* (3)

*θ = (T-T_surr_)/ (T_max_ -T_surr_)* (4)

where *τ_s_* is the sample system time constant; *m* and *C_p_* are the mass (1.0 g) and the thermal capacity (pure water data of 4.2 J·g^-1^·℃^-1^ was used); *T* is the temperature at the cooling time (*t*) after turning off the light source. By linear fitting cooling time (*t*) to negative natural logarithm of temperature (-*ln θ*), *τ_s_* was calculated to be 257 s for DiR Sol with the concentration of 0.3 mg/mL.

The value of *Q_dis_* was determined from the control experiment of pure water using the following equation:

*Q_dis_ = h_0_S(T_max,water_-T_surr,water_)* (5)

*Qdis* was determined to be 0.01 W. Thus, the *η* value was calculated to be 30 % for DiR Sol. The η value of DG NPs and DG PEG_2K_ NPs was measured and calculated by the same method. (31.2% for DG NPs and 31.7% for DG PEG_2K_ NPs).

**Additional Figures**

**Fig S1.** *In vitro* synergistic cytotoxicity (combination index, CI) at various dose ratios of DiR and GA ranging from 1:1 to 10:1.


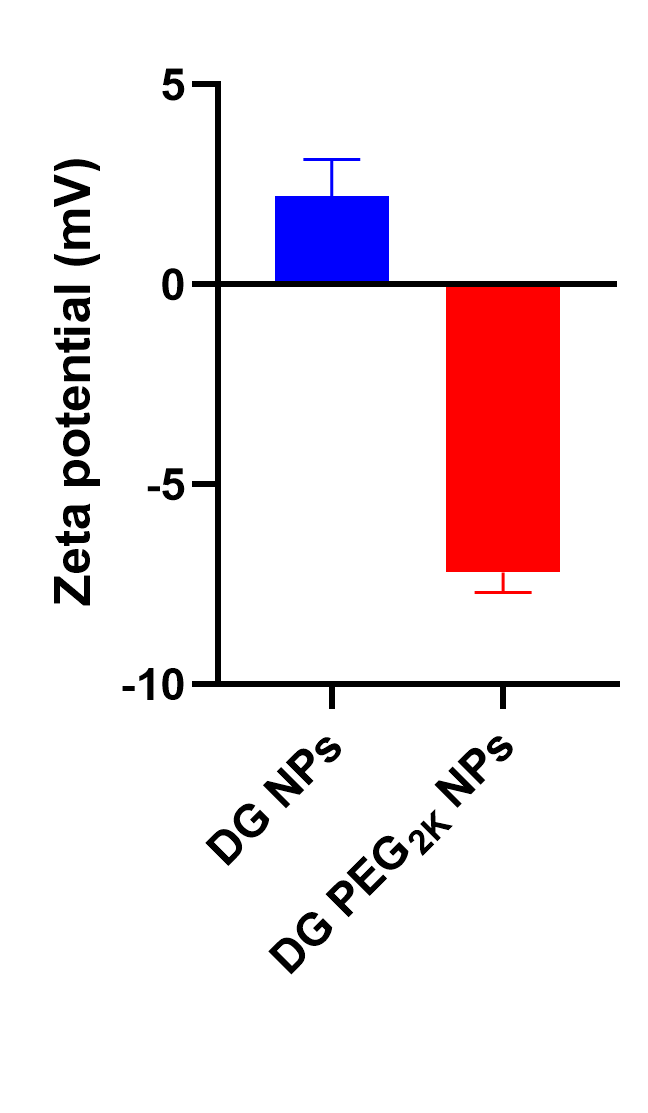


**Fig S2.** Zeta potentials of DG NPs and DG PEG_2K_ NPs.

**Fig S3.** Particle size changes of DG NPs treated with urea, NaCl or SDS (100 mM).


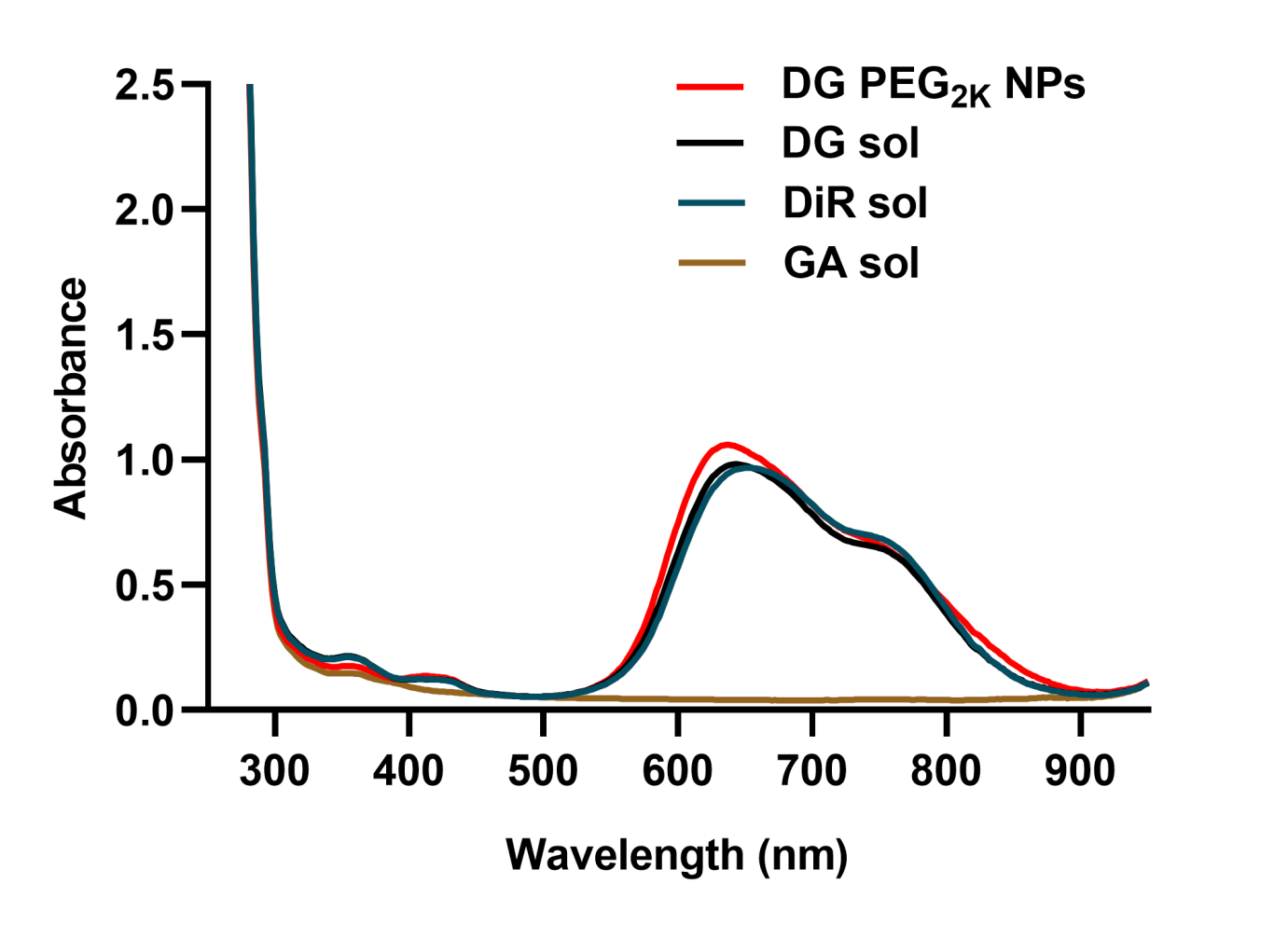


**Fig S4.** UV absorption spectra of DG PEG_2K_ NPs, DG Sol, DiR Sol and GA Sol at 250-950 nm.


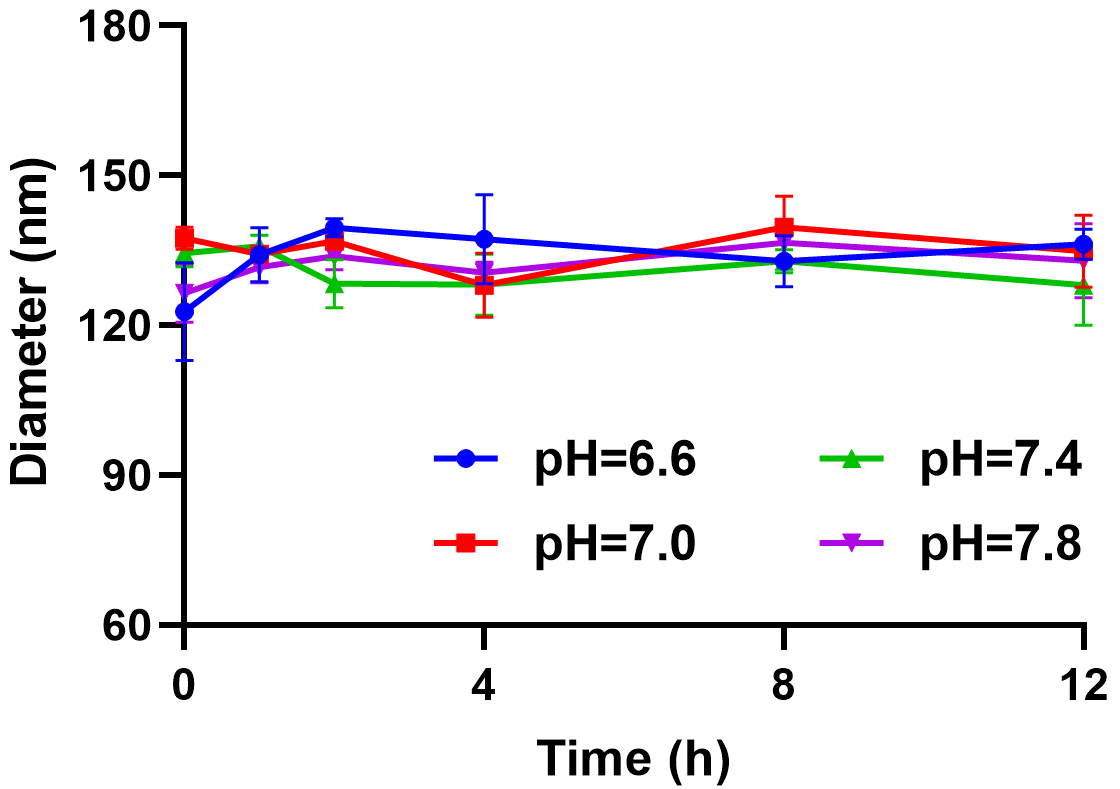


**Fig S5.** Colloidal stability of DG PEG_2K_ NPs during incubation in 10% FBS supplemented PBS at pH of 6.6, 7.0, 7.4 and 7.8 (n=3).

**
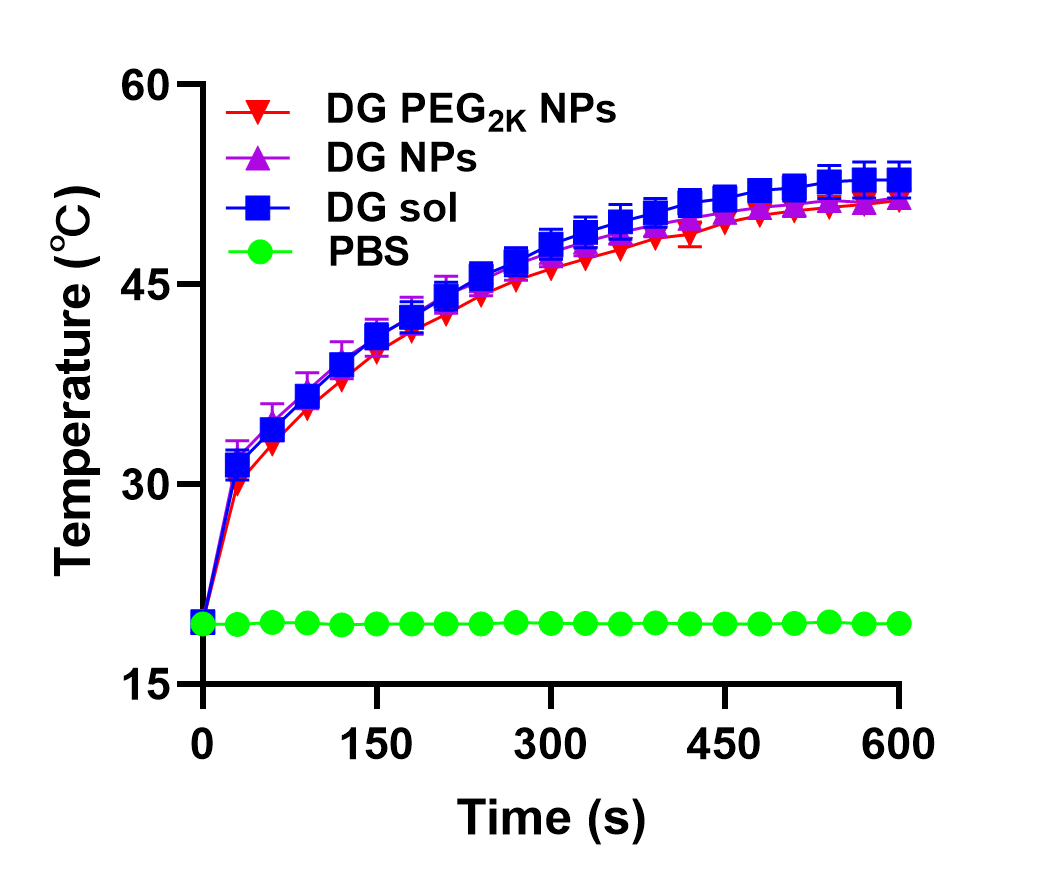
**

**Fig S6.** *In vitro* photothermal efficiency of DG Sol, DG NPs and DG PEG_2K_ NPs in deionized water (n=3).


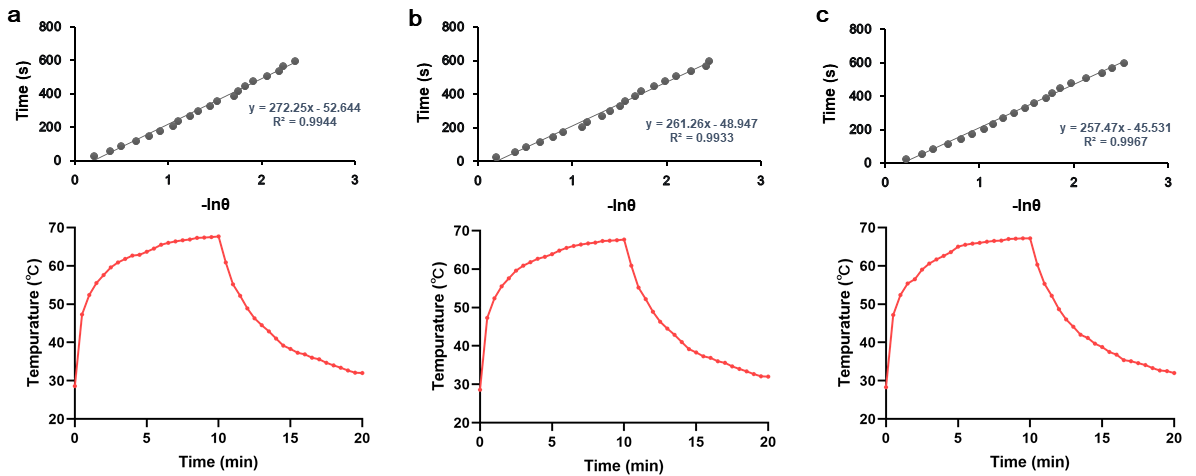


**Fig S7.** PCE of DiR Sol and nanoassemblies irradiated with an 808 nm laser (2 W/cm^2^) for 10 min and then turned off the light source to cool sample for 10 min. The temperature versus time curve was recorded. (a) PCE of DiR Sol; (b) PCE of DG NPs and (c) PCE of DG PEG_2K_ NPs. The standard curve in each figure shows the time versus the negative natural logarithm of the temperature from the cooling test.

**Fig S8.** Cumulative release of GA from DG PEG_2K_ NPs with or without laser irradiation (808 nm, 3 W/cm^2^，5 min).


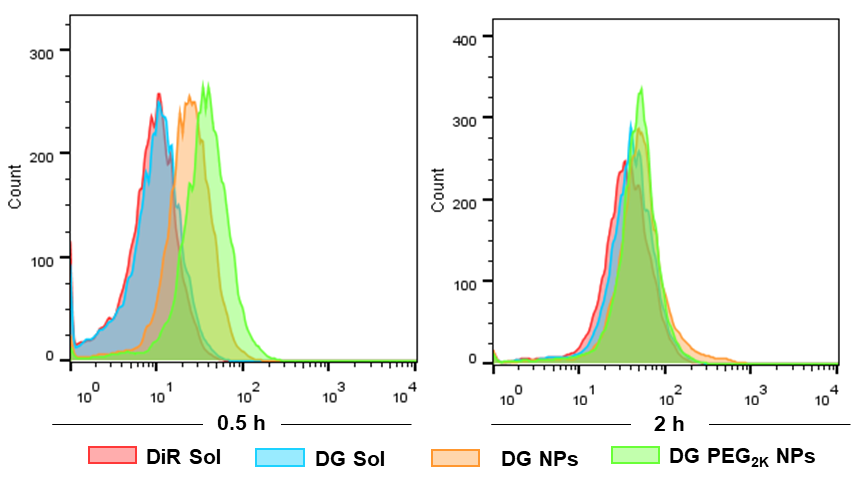


**Fig S9.** Flow cytometry of cellular uptake in 0.5 and 2 h.


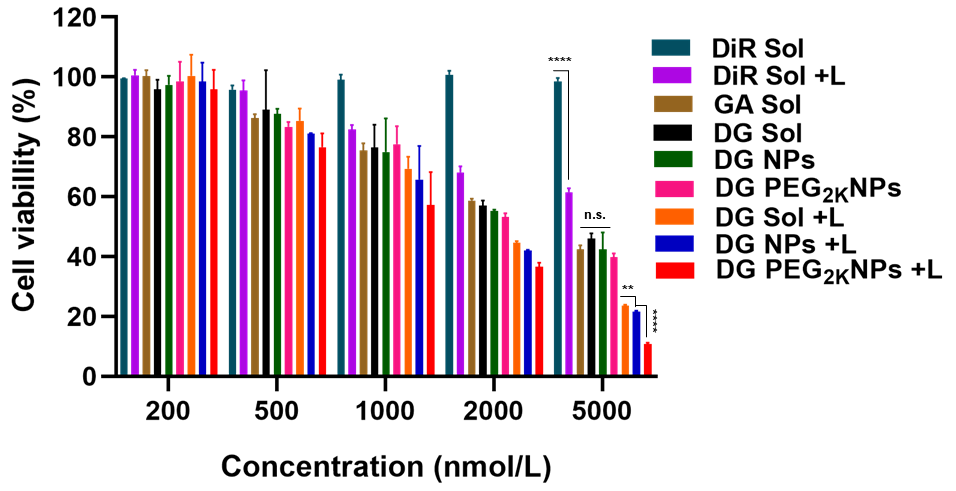


**Fig S10.** Synergistic cytotoxicity against 4T1 cells under 808 nm laser (3 W/cm^2^, 3 min) **P<0.01， **** P < 0.0001, n.s. no significance.


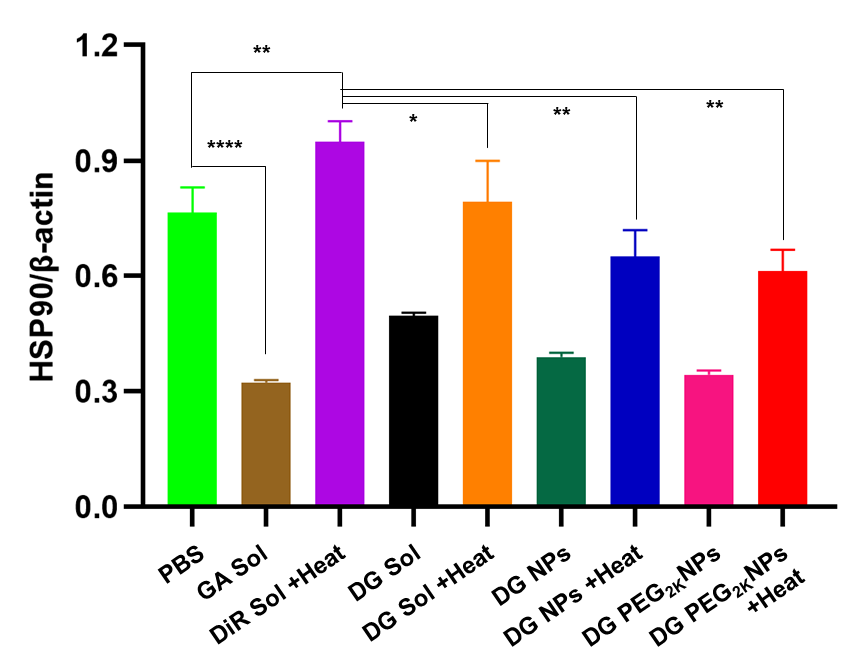


**Fig S11.** Relative expression rates of HSP90 quantified by grayscale analysis. *P<0.05, **P<0.01, **** P < 0.0001.


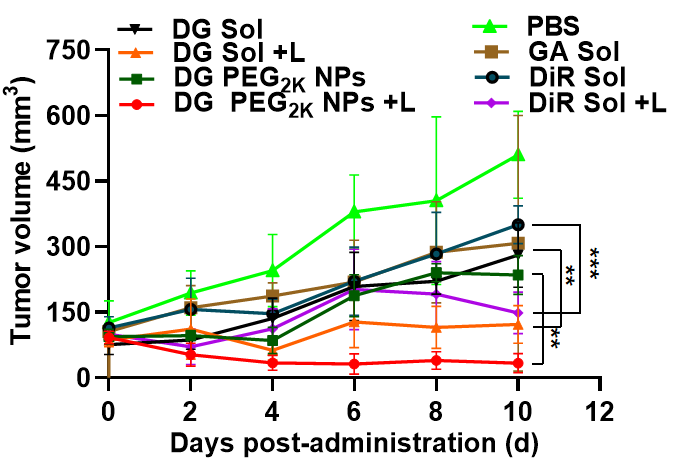


**Fig S12.** Tumor growth profiles on 4T1 tumor bearing mice during therapeutic cycle. ** = P < 0.01, *** = P < 0.001.


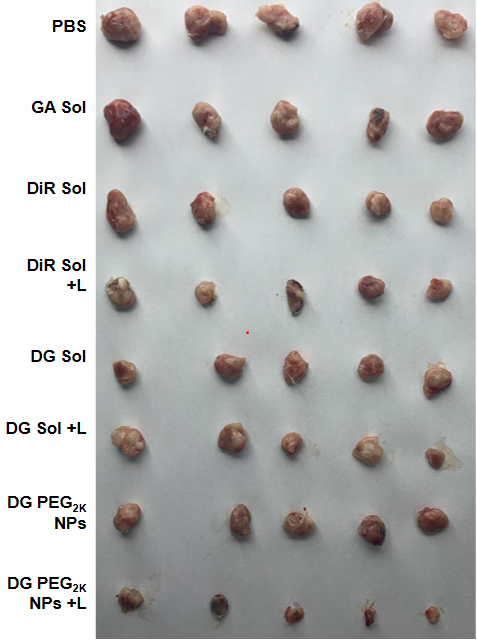


**Fig S13.** *Ex vivo* tumor image after final treatment.


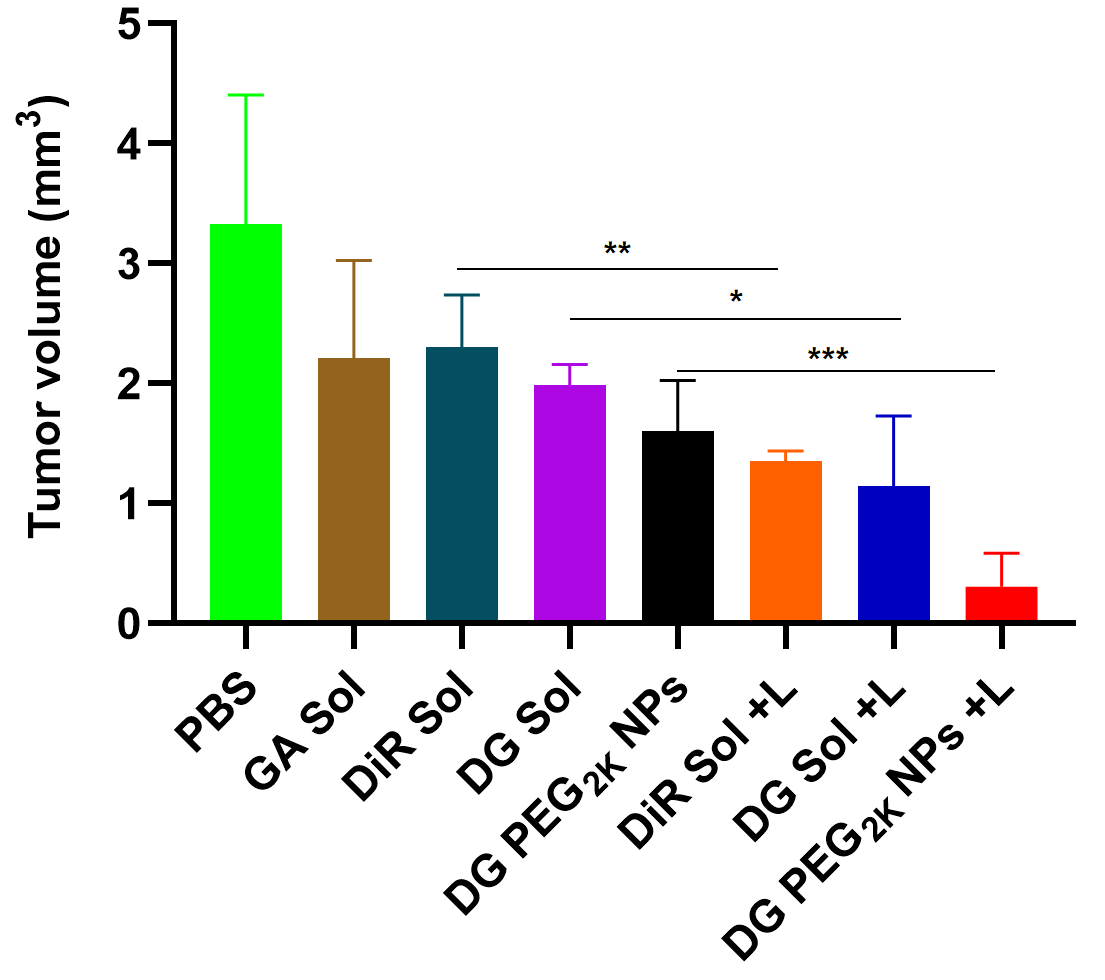


**Fig S14.** Tumor % of body weight of each group after final treatment.


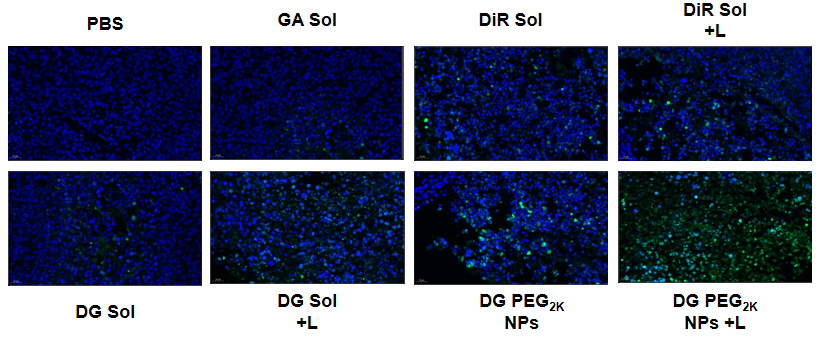


**Fig S15**. Immunofluorescence staining of TUNEL assay.


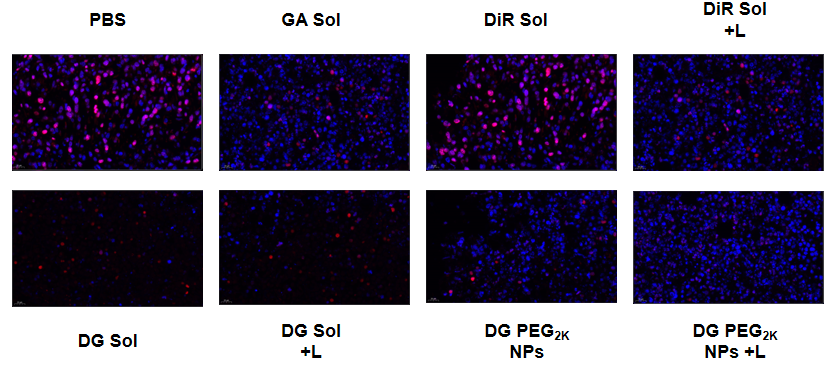


**Fig S16.** Immunofluorescence staining of Ki67.


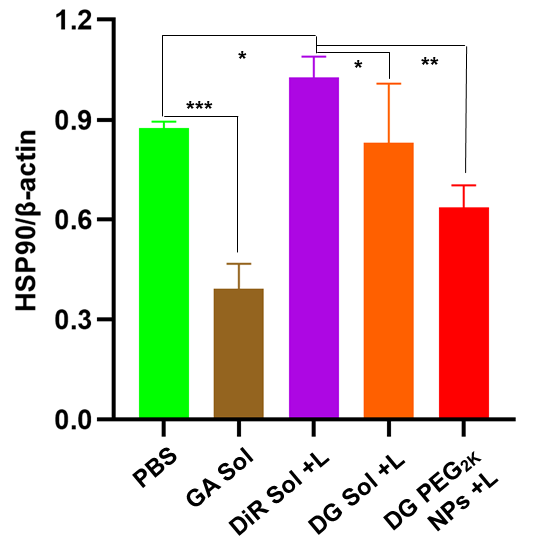


**Figure S17.** Relative expression rates of HSP90 quantified by grayscale analysis.


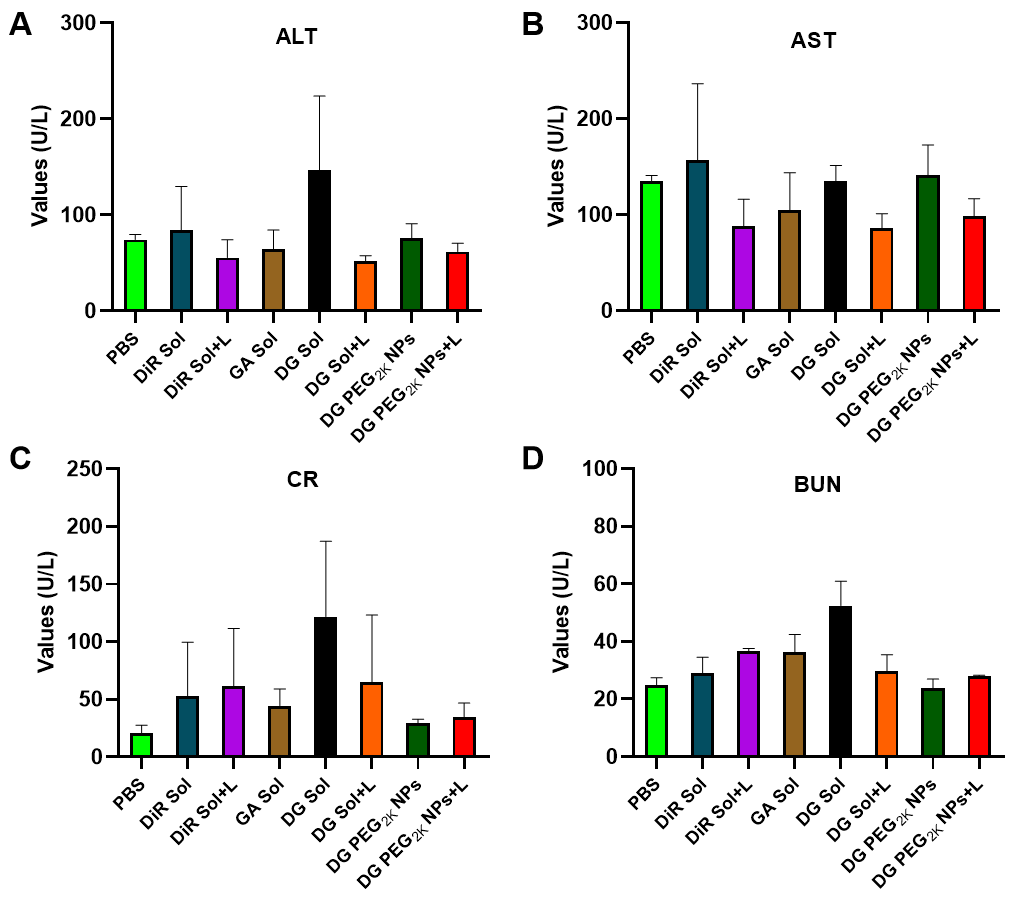


**Fig S18.** Hepatorenal function indicators of 4T1 tumor-bearing mice after treatment. (n=3). (A) ALT: alanine aminotransferase; (B) AST: aspartate aminotransferase; (C) BUN: blood urea nitrogen; (D) CREA: creatinine.


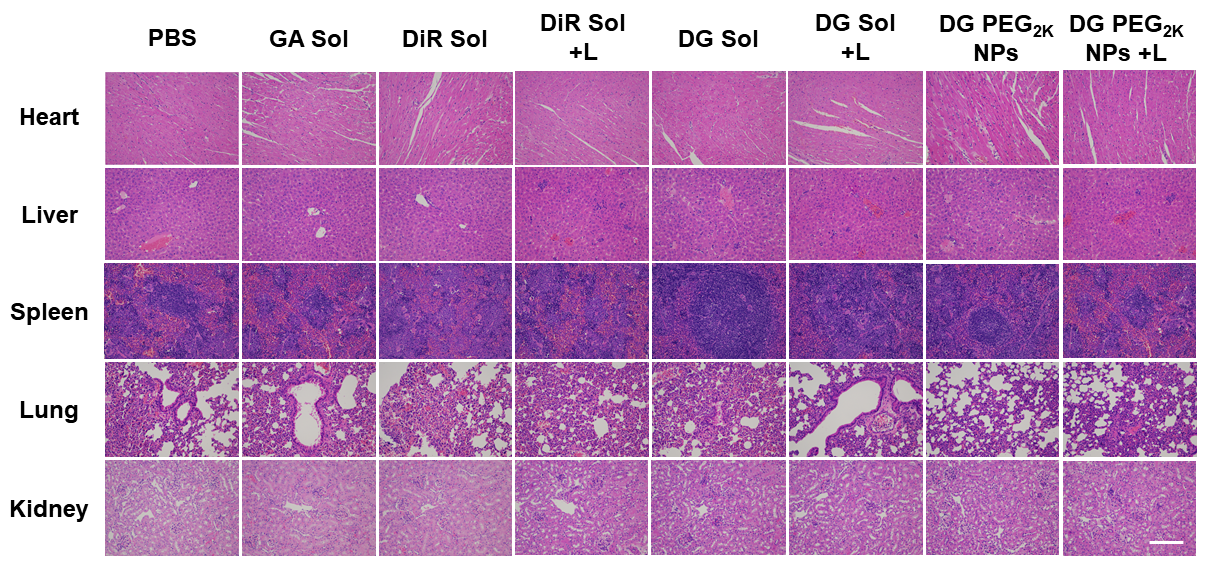


**Fig S19.** H&E staining images of heart, lung, spleen, lung and kidney after different treatments. H&E staining: 200 × magnification. Scale bar= 100 μm.

**Additional Tables**

**Tab. S1.** Characterization of non-PEGylated hybrid nanoassemblies with various molar ratios.

| DiR:GA | ^a)^ Size (nm) | ^b)^ PDI |
| --- | --- | --- |
| 1:1 | 185.1 ± 4.2 | 0.150 ± 0.04 |
| 2:1 | 91.53 ± 1.7 | 0.163 ± 0.07 |
| 3:1 | 93.25 ± 1.9 | 0.205 ± 0.02 |
| 5:1 | 78.73 ± 3.0 | 0.167 ± 0.09 |
| 7:1 | 85.36 ± 0.50 | 0.194 ± 0.04 |
| 10:1 | 89.45 ± 2.7 | 0.210 ± 0.02 |

^a)^ Mean diameters of nanoassemblies were determined by DLS. ^b)^ Polydispersity index of particles size.

**Tab. S2.** Characterization of PEGylated hybrid nanoassemblies with various molar ratios.

| DiR:GA | ^a)^ Size (nm) | ^b)^ PDI |
| --- | --- | --- |
| 1:1 | 107.4 ± 3.4 | 0.098 ± 0.03 |
| 2:1 | 75.57 ± 0.85 | 0.207 ± 0.04 |
| 3:1 | 89.41 ± 3.6 | 0.128 ± 0.11 |
| 5:1 | 89.50 ± 2.5 | 0.220 ± 0.01 |
| 7: 1 | 115.8 ± 1.8 | 0.101 ± 0.03 |
| 10:1 | 93.25 ± 1.5 | 0.144 ± 0.04 |

^a)^ Mean diameters of nanoassemblies were determined by DLS. ^b)^ Polydispersity index of particles size.

**Tab. S3.** Optimization of molar ratios of co-nanoassembly.

| DiR:GA | ^a)^ IC_50_ (μM) | ^b)^ CI index |
| --- | --- | --- |
| 1:1 | 1.42 ± 0.01 | 0.56 |
| 2:1 | 1.64 ± 0.01 | 0.51 |
| 3:1 | 1.87 ± 0.01 | 0.37 |
| 5:1 | 4.10 ± 0.10 | 0.54 |
| 7:1 | 6.23 ± 0.18 | 0.63 |
| 10:1 | 9.36 ± 0.18 | 0.71 |

^a)^ IC_50_ value on several proportions of DiR and GA; ^b)^ Combination index (CI) on several proportions of DiR and GA calculated by IC_50_ values.

**Tab. S4.** Characterization of DG NPs and DG PEG_2K_ NPs.

| Nanoassemblies | ^a)^ Size (nm) | ^b)^ PDI | ^c)^DL_DiR_ | ^c)^DL_GA_ |
| --- | --- | --- | --- | --- |
| DG NPs | 93.25 ± 1.9 | 0.205 ± 0.11 | 82.7% | 17.3% |
| DG PEG_2K_ NPs | 89.41 ± 3.6 | 0.128 ± 0.11 | 66.1% | 13.9% |

^a)^ Mean diameters were determined by DLS. ^b)^ Polydispersity index of particles size. ^c)^ Drug-loading of DiR or GA was calculated by the molecular weight of drugs and the amount of DSPE-PEG_2K_.

**Tab. S5.** *In vitro* cytotoxicity (IC_50_ values) of DiR and GA nanoassemblies to 4T1 cells. (MTT assay).

| Formulations | IC_50_ (μM) | |
| --- | --- | --- |
|  | Laser (+) | Laser (-) |
| GA Sol | - | 3.27 ± 0.18 |
| DiR Sol | 6.22 ± 0.07 | 33.86 ± 22.25 |
| DG mixture Sol | 1.99 ± 0.17 | 3.43 ± 0.52 |
| DG NPs | 1.74 ± 0.26 | 3.08 ± 0.46 |
| DG PEG_2K_ NPs | 1.29 ± 0.13 | 2.90 ± 0.99 |

**Additional Reference**

1. Roper DK, Ahn W, Hoepfner M: **Microscale Heat Transfer Transduced by Surface Plasmon Resonant Gold Nanoparticles.** *J Phys Chem C Nanomater Interfaces* 2007, **111:**3636-3641.
